# Prevalence of and characteristics associated with insomnia and obstructive sleep apnea among veterans with knee and hip osteoarthritis

**DOI:** 10.1186/s12891-018-1993-y

**Published:** 2018-03-09

**Authors:** Shannon Stark Taylor, Jaime M. Hughes, Cynthia J. Coffman, Amy S. Jeffreys, Christi S. Ulmer, Eugene Z. Oddone, Hayden B. Bosworth, William S. Yancy, Kelli D. Allen

**Affiliations:** 1 University of South Carolina School of Medicine Greenville, 607 Grove Rd, Greenville, SC 29605 USA; 2 Durham VA Health Care System, VA Medical Center (152), 508 Fulton Street, Durham, NC 27705 USA; 3 Duke University School of Medicine, Durham, NC USA; 4 University of North Carolina at Chapel Hill, Chapel Hill, NC USA

**Keywords:** Osteoarthritis, Sleep disturbance, Insomnia, Obstructive sleep apnea, Chronic pain, Veterans

## Abstract

**Background:**

Few studies have examined patterns of specific sleep problems among individuals with osteoarthritis (OA). The primary objective of this study was to examine prevalence of symptoms of insomnia and obstructive sleep apnea (OSA) among Veterans with OA. Secondary objectives were to assess proportions of individuals with insomnia and OSA symptoms who may have been undiagnosed and to examine Veterans’ characteristics associated with insomnia and OSA symptoms.

**Methods:**

Veterans (*n* = 300) enrolled in a clinical trial completed the Insomnia Severity Index (ISI) and the Berlin Questionnaire (BQ) at baseline; proportions of participants with symptoms consistent with insomnia and OSA were calculated, using standard cut-offs for ISI and BQ. For Veterans with insomnia and OSA symptoms, electronic medical records were searched to identify whether there was a diagnosis code for these conditions. Multivariable linear (ISI) and logistic (BQ) regression models examined associations of the following characteristics with symptoms of insomnia and OSA: age, gender, race, self-reported general health, body mass index (BMI), diagnosis of post-traumatic stress disorder (PTSD), pain severity, depressive symptoms, number of joints with arthritis symptoms and opioid use.

**Results:**

Symptoms consistent with insomnia and OSA were found in 53 and 66% of this sample, respectively. Among participants screening positive for insomnia and OSA, diagnosis codes for these disorders were present in the electronic medical record for 22 and 51%, respectively. Characteristics associated with insomnia were lower age (β (SE) = − 0.09 (0.04), 95% confidence interval [CI] = − 0.16, − 0.02), having a PTSD diagnosis (β (SE) = 1.68 (0.73), CI = 0.25, 3.11), greater pain severity (β (SE) = 0.36 (0.09), CI = 0.17, 0.55), and greater depressive symptoms (β (SE) = 0.84 (0.07), CI = 0.70, 0.98). Characteristics associated with OSA were higher BMI (odds ratio [OR] = 1.13, CI = 1.06, 1.21), greater depressive symptoms (OR = 1.12, CI = 1.05, 1.20), and opioid use (OR = 0.51, CI = 0.26, 0.99).

**Conclusions:**

Insomnia and OSA symptoms were very common in Veterans with OA, and a substantial proportion of individuals with symptoms may have been undiagnosed. Characteristics associated with insomnia and OSA symptoms were consistent with prior studies.

**Trial registration:**

NCT01130740.

## Background

There is increasing recognition that sleep disturbance is a common comorbidity among individuals with osteoarthritis (OA) [1–4]. Previous studies suggest that at least half of patients with OA report significant sleep disturbance [4–6], with some studies indicating the prevalence may be as high as 70% [1, 7]. Common sleep disturbances include insomnia and obstructive sleep apnea (OSA). Insomnia is marked by difficulty falling asleep, staying asleep, or waking too early, and these difficulties may contribute to daytime impairment. OSA is characterized by brief cessations in breathing which cause sleep to be fragmented and non-restorative. Sleep problems can have a negative impact on many outcomes, including psychological health, quality of life, and cardiovascular morbidity and mortality [8, 9]. Among patients with OA, sleep problems are also associated with greater pain severity [4]. This relationship is likely a reciprocal one; in some studies sleep disturbance has predicted subsequent increases in pain severity, and in others, pain has predicted subsequent sleep disturbance [10].

Few studies have examined factors associated with sleep problems among patients with OA [1, 6]. Prior findings suggest that in addition to greater pain severity, other factors associated with worse sleep quality include: a greater number of arthritic joints, depression, poor overall health, reduced physical function, less social support, and less education [1, 6]. These studies evaluated overall sleep quality, but interestingly, there has been very little study of the prevalence of OSA or factors associated with OSA among patients with OA. The prevalence of OSA is elevated in patients with other rheumatic conditions [11], and because many patients with OA are overweight or obese – a main risk factor for OSA – it seems likely that rates would be elevated among these patients as well. In one study of 254 patients awaiting hip or knee arthroplasty, 6.7% of patients had OSA based on polysomnography (overnight sleep study). Importantly, the majority of these cases were undiagnosed. A recent analysis of a population-based cohort of men did not find links between OSA and the presence of musculoskeletal joint pain or the number of painful joints, although OA diagnosis was not specifically assessed [12]. However, in a study of patients referred for polysomnography, there was a strong association between OSA severity and OA severity, independent of body mass index [13]. The primary objective of this study was to examine the prevalence of OSA and insomnia symptoms among individuals with hip and knee OA. Secondary objectives were to: 1) examine participant characteristics associated with OSA and insomnia symptoms, 2) assess proportions of individuals with OSA and insomnia symptoms who may have been undiagnosed (based on information in the electronic medical record), 3) examine participant characteristics associated with under-diagnosis.

## Methods

### Study design

Data for these cross-sectional analyses were from baseline assessments collected in the study of Patient and Provider Interventions for Managing Osteoarthritis in Primary Care (PRIMO). Details of this study are described elsewhere [14, 15]. Briefly, this was a cluster-randomized controlled trial, with primary care providers (PCPs) assigned to two study arms: OA Intervention and Usual Care control. The institutional review board of the Department of Veterans Affairs HealthCare System in Durham, NC (DVAHCS) approved this study. All procedures performed were in accordance with the ethical standards of the institutional research committee and with the 1964 Helsinki declaration and its later amendments. Written informed consent was obtained from all participants.

### Participants

Participants (*N* = 300) were patients at Durham VA Medical Center who had symptomatic hip OA (based on radiographic evidence in the electronic medical record) and/or symptomatic knee OA (based on radiographic evidence in the electronic medical record or meeting American College of Rheumatology clinical criteria) [16]. Participants were also overweight or obese (body mass index (BMI) ≥ 25) and not currently meeting weekly physical activity guidelines set forth by the Department of Health and Human Services (DHHS) [17]. Additional information regarding exclusion criteria and recruitment processes are provided elsewhere [14].

### Measures

#### Insomnia

Insomnia symptoms were measured with the Insomnia Severity Index (ISI). The scale content corresponds to DSM-IV criteria for insomnia and measures respondents’ perceptions of symptoms during the past 2 weeks. Scores were analyzed on a continuous scale with higher scores indicating greater insomnia symptom severity. In addition, a dichotomous indicator of symptoms consistent with insomnia was calculated, based on a score greater than 10 on the 0–28 scale [18].

#### Sleep apnea

OSA risk was assessed using the Berlin Questionnaire (BQ) [19], an11-item measure based on frequency and intensity of snoring, frequency of daytime sleepiness or fatigue, and presence of obesity or hypertension. Respondents are considered “high risk” for OSA if they have both persistent snoring and daytime fatigue in combination with a diagnosis of hypertension or elevated BMI (≥30 kg/m^2^) [20]. The sensitivity ranges from 76 to 84% and the specificity ranges from 38 to 59% among individuals in the general population. Among primary care patients, the sensitivity ranges from 54 to 86% and the specificity ranges from 43 to 87% [19].

#### Insomnia and OSA diagnoses

VA electronic medical records were reviewed to ascertain if a diagnosis of insomnia and/or OSA had ever been recorded for each participant. ICD-9 codes for insomnia were 780.52, 307.42, 327.02, 327.01, 307.41, 327.09, 46.72, 327, 292.85, 291.82, 780.51 and for sleep apnea were 786.03, 327.23, 780.53, 70.57, 327.21, 327.29, 327.2, 327.27, 780.51.

#### Patient demographic and clinical characteristics

We identified demographic and clinical characteristics with potential links to sleep disturbance based on prior studies [1, 6]. We collected self-reported information on age, sex, race/ethnicity (white vs. nonwhite), self-rated health (excellent, very good, or good vs. fair or poor), and number of joints with OA. Body mass index was calculated using measured height and weight collected at the baseline assessment.

Pain was measured using the 5-item subscale from the Western Ontario and McMaster Universities Osteoarthritis Index (WOMAC), a self-reported measure of lower extremity pain in the previous 2 weeks [21–23]. All items are rated on a 5-point Likert scale ranging from “none” to “extreme”; scores range from 0 to 25, with higher scores indicating worse symptoms. Depressive symptoms were assessed with the Patient Health Questionnaire (PHQ-8), a reliable and valid measure [24]. Participants rated the extent to which they were bothered by a series of complaints based on the DSM-5 criteria for Major Depressive Disorder over the prior 2 weeks. Each of the 8 questions is scored as 0 (not at all) to 3 (nearly every day), so that total scores range from 0 to 24, with higher scores indicating greater depressive symptom severity. PTSD diagnosis was ascertained using a data pull to identify patients having ever received a PTSD diagnosis based on ICD-9 coding (code 309.81) in the electronic medical record. Opioid use was assessed because of its potential contributions to sleep problems. Participants were asked to bring all of their current pain-related medications to the baseline visit; the research assistant documented the class of each medication, and a dichotomous variable was created to indicate whether participants were using opioids at the time of study entry.

### Statistical analysis

Descriptive statistics, including means and standard deviations (SD) for continuous variables and frequencies for categorical variables, were calculated. Bivariate linear (insomnia scores on ISI) and logistic (dichotomous OSA risk from BQ) regression models were fit separately for each patient characteristic variable described above. We then fit multivariable linear (ISI) and logistic (BQ) regression models including all patient characteristic variables to examine the associations of patient and clinical characteristics with each condition. In the subgroup of participants that met self-reported criteria for insomnia or OSA, multivariable logistic regression models were fit to examine the association of each patient characteristic with the presence of ICD-9 codes for OSA or insomnia in the VA medical record. Residual plots from models were examined to assess normality assumptions, and goodness of fit was examined for logistic regression models. Linearity assumptions for the continuous covariates in all models were assessed and found not to have been violated. Data management and analyses were conducted in SAS version 9.4 (SAS Institute, Cary, NC).

## Results

Participants were an average age of 61.1 (SD = 9.2) years, predominantly male (90.7%), and obese (mean BMI 33.8; SD = 5.8). Additional participant characteristics are shown in Table 1.

**Table 1:** Participant demographic and clinical characteristics

Participant Characteristics (*n* = 300)	M (SD)/ % (N)
Age	61.1 (9.2)
Gender	
Male	91% (273)
Race	
Non-Hispanic White	50% (150)
Education	
Some College Education	73% (219)
Self-Rated Health	
Excellent, Very Good, or Good	62% (186)
BMI	33.8 (5.9)
PTSD Diagnosis	28% (84)
WOMAC Pain^a^	10.2 (4.0)
Depressive Symptoms^b^	6.8 (5.4)
# Joints with Arthritis Symptoms	6.2 (3.6)
Opioid Use	30% (88)
Years with OA Symptoms	14.5 (12.6)
Insomnia severity	
ISI	11.4 (8.0)
Obstructive sleep apnea risk	
High risk per BQ	66% (198)

*BMI* body mass index, *PTSD* post-traumatic stress disorder, *WOMAC* Western Ontario and McMaster Universities Osteoarthritis Index, *OSA* obstructive sleep apnea

^a^Possible Range = 0–20

^b^Possible Range = 0–24

2 participants are missing ISI and Berlin scores, 1 participant is missing a WOMAC pain score, and 4 patients are missing a PHQ-8 score. 4 participants are missing data on opioid use

### Descriptive characteristics of insomnia and OSA

**Fig. 1:**
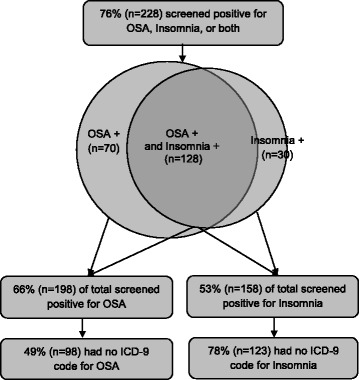
Visual representation of frequencies of individuals screening positive for OSA, insomnia, or both. Two individuals had missing ISI scores and two individuals had missing BQ scores

Frequencies and descriptive characteristics for the ISI and BQ and medical record diagnosis codes are presented in Fig. 1. Seventy-six percent of participants (*n* = 228/300) screened positive for at least one sleep disturbance, including 23% screening “high risk” for sleep apnea alone (*n* = 70), and 10% screening positive for insomnia alone (*n* = 30). Forty-two percent (*n* = 128) screened positive for both insomnia and OSA. More than half of the sample (53%; *n* = 158/300) screened positive for insomnia, but only 22% (*n* = 35) of those individuals had an ICD-9 code for insomnia in the medical record. Although more than 66% of the sample (*n* = 198/300) screened in the “high risk” category for OSA, only about half of these (51%; *n* = 101) had an ICD-9 code for OSA in the medical record.

### Demographic and clinical factors associated with insomnia severity

In bivariate models, higher self-reported insomnia severity was related to younger age, non-white race, poorer self-rated health, a diagnosis of PTSD in the medical record, higher self-reported arthritis pain, more self-reported depressive symptoms, more joints with arthritis, and current opioid use (Table 2). In the multivariable linear model, younger age (Estimate = − 0.09, SE = 0.04, 95% Confidence Interval (CI) = − 0.16, − 0.02, *p* = .01), PTSD diagnosis (Estimate = 1.68, SE = 0.73, CI = 0.25, 3.11, *p* = .02), pain (Estimate = 0.36, SE = 0.10, CI = 0.17, 0.55, *p* < .001), and depressive symptoms (Estimate = 0.84, SE = 0.07, CI = 0.70, 0.98, *p* < .001), were associated with insomnia severity (Table 2).

**Table 2:** Bivariate and Multivariable Linear Regression Models of Insomnia (ISI) Scores

Bivariate and Multivariable Linear Regression Models of Insomnia (ISI) Scores						
	Bivariate Model			Multivariable Model		
	Estimate (SE)	95% CI	*p*	Estimate (SE)	95% CI	*p*
Age	−0.29 (0.05)	− 0.38, − 0.19	<.001	−0.09 (0.04)	− 0.16, − 0.02	.01
Gender						
Male	1.34 (1.59)	−1.78, 4.46	.40	−0.20 (1.07)	−2.30, 1.89	.85
Race						
Non-Hispanic White	−3.07 (0.91)	−4.86, −1.28	.001	−0.19 (0.65)	−1.47, 1.08	.76
Self-Rated Health						
Excellent, Very Good, or Good	−5.82 (0.89)	−7.58, −4.07	<.001	−0.82 (0.73)	−2.26, 0.62	.26
BMI	0.11 (0.08)	−0.04, 0.27	.15	−0.06 (0.06)	−0.17, 0.06	.34
PTSD Diagnosis	5.65 (0.98)	3.73, 7.57	<.001	1.68 (0.73)	0.25, 3.11	.02
WOMAC Pain	1.05 (0.10)	0.85, 1.25	<.001	0.36 (0.10)	0.17, 0.55	<.001
Depressive Symptoms						
PHQ-8	1.10 (0.06)	0.99, 1.22	.001	0.84 (0.07)	0.70, 0.98	<.001
# Joints with Arthritis	0.49 (0.12)	0.25, 0.74	< 0.001	0.07 (0.09)	−0.11, 0.25	.44
Opioid Use	3.38 (0.99)	1.42, 5.33	< 0.001	0.18 (0.71)	−1.23, 1.58	0.81

All bivariate models had 2 missing observations. Otherwise, the unadjusted model with the model with WOMAC Pain has 1 additional missing observation and the model with PHQ-8 and the model with opioid use have 4 additional missing observations. The multivariable model has 11 missing observations. Negative scores associated with less insomnia symptoms. Higher WOMAC scores indicate greater pain. Self-rated health was recoded into a binary variable: excellent, very good, and good health vs. fair/poor health

### Demographic and clinical factors associated with OSA risk

In bivariate logistic models, the odds of high risk of OSA were higher for younger age, fair or poor self-rated health, higher BMI, diagnosed with PTSD, higher self-reported arthritis pain, higher reporting of depressive symptoms, and reporting more joints with arthritis (Table 3). In the multivariable logistic model, BMI (Odds ratio (OR) = 1.1, CI = 1.06, 1.21, *p* < .001), depressive symptoms (OR = 1.12, CI = 1.05, 1.20, *p* = .002), and opioid use (OR = 0.51, CI = 0.26, 0.99, *p* = .05) were associated with high risk for OSA (Table 3).

**Table 3:** Bivariate and Multivariable Models of “High Risk” for Sleep Apnea (BQ)

Bivariate and Multivariable Models of “High Risk” for Sleep Apnea (BQ)								
	Low Risk	High Risk	Bivariate Model			Multivariable Model		
	*N* = 102	N = 198						
	M (SD)/ % (N)	M (SD)/ % (N)	OR	95% CI	*p*	OR	95% CI	*p*
Age	63.40 (9.47)	59.76 (8.74)	0.96	0.93, 0.98	.002	0.97	0.94, 1.00	.08
Gender								
Male	88% (88)	92% (182)	1.55	0.70, 3.42	.28	2.46	0.94, 6.43	.07
Race								
Non-Hispanic White	52% (52)	49% (97)	0.89	0.55, 1.43	.62	1.52	0.83, 2.76	.18
Self-Rated Health								
Excellent, Very Good, or Good	76% (78)	54% (107)	0.38	0.22, 0.65	<.001	0.60	0.30, 1.18	.14
BMI	31.93 (4.46)	34.8 (6.23)	1.11	1.06, 1.17	<.001	1.13	1.06, 1.21	<.001
PTSD Diagnosis	18% (18)	34% (67)	2.33	1.29, 4.20	.005	1.84	0.90, 3.75	.09
WOMAC Pain	9.13 (3.77)	10.73 (3.90)	1.11	1.04, 1.19	.001	0.99	0.91, 1.08	.83
Depressive Symptoms								
PHQ-8	4.32 (4.25)	7.94 (5.37)	1.17	1.10, 1.24	<.001	1.12	1.05, 1.20	.002
# Joints with Arthritis	5.54 (3.14)	6.52 (3.70)	1.09	1.01, 1.17	0.03	1.07	0.98, 1.17	.13
Opioid Use	29% (28)	30% (60)	1.08	0.63, 1.84	0.78	0.51	0.26, 0.99	.05

All unadjusted models have 2 missing values because 2 participants did not complete the BQ. Otherwise, the unadjusted model with the model with WOMAC Pain has 1 additional missing observation and the model with PHQ-8 and the model with opioid use have 4 additional missing observations. The multivariable model has 11 missing observations. Higher WOMAC scores indicate greater pain. Self-rated health was recoded into a binary variable: excellent, very good, and good health vs. fair/poor health

### Demographic and clinical factors associated with documented insomnia and OSA diagnoses

Among participants (*n* = 158) with a positive screen for insomnia, none of the patient characteristics examined in bivariate analyses (ORs < 1.48; *p*’s > .10) or the multivariable model (ORs < 1.47; *p*’s > .10) were associated with the presence of a diagnosis code in the medical record.

Among participants (*N* = 198) with a positive screen for OSA, in bivariate logistic models the following factors were associated with an OSA ICD-9 code in the medical record: poorer self-rated health (OR = 0.46, CI = 0.26, 0.80, *p* = .007), higher BMI (OR = 1.08, CI = 1.03, 1.14, *p* = .002), a diagnosis of PTSD (OR = 2.83, CI =1.53, 5.24, *p* = .001), more arthritis-related pain (OR = 1.08, CI = 1.00, 1.16, *p* = .05), more symptoms of depression (OR = 1.10, CI = 1.04, 1.16, *p* < .001), and opioid use (OR = 2.09, CI = 1.12, 3.90, *p* = .02). In multivariable models, among participants with a positive screen for OSA (BQ “high risk”), the odds of having with a diagnosis code in the medical record was greater for higher BMI (OR = 1.11, CI = 1.04,1.18, *p* < .001), a diagnosis of PTSD in the medical record (OR = 2.51, CI = 1.24, 5.07, *p* = .01), and more self-reported depressive symptoms (OR = 1.08, CI = 1.00, 1.16, *p* = .04).

## Discussion

Self-reported symptoms consistent with insomnia and OSA were highly prevalent in this cohort, with a high degree of overlap between those screening positive for both conditions. Patient characteristics associated with insomnia and OSA were largely consistent with prior studies. A key finding was that many individuals with insomnia and OSA symptoms did not have corresponding diagnosis codes in the medical record, potentially indicating substantial under-diagnosis. This illustrates the need for more consistent screening for insomnia and OSA, given numerous negative consequences of untreated sleep problems [8, 9].

Fifty-two percent of this sample screened positive for insomnia, which is notably higher than the estimated prevalence of insomnia in the general population, (18%) and in adults aged 55–64 (24%) [25]. This finding is consistent with higher reported prevalence of insomnia in Veterans in other studies, with estimates ranging from 24 to 54% [26]. Although a higher prevalence of insomnia has also been reported in individuals with OA (23%) compared to the general population [2], the prevalence in this sample was still much higher. Overall, our results are consistent with previous studies demonstrating links of insomnia with PTSD diagnosis, pain, and depressive symptoms in the general population [2, 27], among individuals with OA (all but PTSD) [1], and in Veterans [26, 28]. Our finding that increased age was associated with a lower likelihood of screening positive for insomnia is consistent with prior research among Veterans [28, 29], though the converse association has been shown in multiple studies of the general population [30, 31].

The 66% prevalence of “high risk” for OSA is much greater than the estimated prevalence of 17% among US men aged 50–70 (which is a similar demographic to our study sample). Veterans and individuals with OA tend to exhibit characteristics associated with higher OSA risk, including older age, higher BMI, and higher rates of medical comorbidity. The majority of Veterans are also male, and many have psychiatric comorbidities, which are additional OSA risk factors [32, 33] Study findings were consistent with past work showing associations of OSA with BMI and depression, including analyses in Veterans and patients referred for total joint arthroplasty [34–36]. BMI-related findings underscore the importance of OSA-targeted questioning when assessing sleep concerns, as multiple analyses have failed to find associations between BMI and general assessments of sleep quality in individuals with OA [1, 5]. An association between OSA and opioid use is also consistent with prior literature [37].

Forty-three percent of our sample screened positive for both insomnia and OSA. A high prevalence (39–58%) of insomnia symptoms have been reported in individuals in the general population with OSA, and between 29 and 67% of individuals with insomnia had symptoms consistent with high OSA risk [38]. High rates of comorbid insomnia and OSA have also been noted in military personnel [39] and Veterans [40], particularly in those with PTSD, who often present with both conditions due to increased sympathetic activation [41, 42]. Symptoms shared by both insomnia and OSA, including fragmented sleep and daytime impairment, may have contributed to the significant degree of screening overlap and thus, the true comorbidity in this sample may be overestimated.

Consistent with the literature for both conditions, our results suggest a significant gap between the presence of insomnia and OSA symptoms and documented diagnoses [43]. Sleep concerns may be minimized or overlooked in primary care visits, particularly when greater attention is devoted to other chronic conditions.. Among Veterans at high risk for OSA in this study, those with, PTSD, higher BMI, and greater depressive symptoms were more likely to have an OSA diagnosis noted in the medical record. This higher rate of documentation in the medical record could be due to more severe sleep disturbance among individuals with these health conditions or because physicians were more likely to screen for or recognize sleep disturbance among individuals with these risk factors [36].

There are some limitations to this study. First, the BQ is not validated for diagnostic use, has a large range of specificity, and may overestimate prevalence in an overweight population. Second, the use of ICD-9 codes may have under-estimated actual physician diagnoses of insomnia and OSA, which may have been documented elsewhere in the medical record. Third, we did not collect patient information about prior treatment for sleep problems (e.g., CPAP), which may have influenced screening scale responses. Fourth, we did not collect information on self-reported PTSD symptoms and therefore relied on the presence of a diagnosis in the medical record, which may not always be an accurate reflection of symptoms. Fifth, we did not obtain de novo radiographs and therefore did not have a measure of radiographic OA severity. Finally, the study population consisted of Veterans (primarily male), with BMIs ≥25, thus, these findings may not generalize across different populations. In particular, this was a group with higher risk for sleep problems than the general population, and patterns of diagnosis may differ outside of the VA healthcare system.

## Conclusions

In summary, this study found high rates of insomnia and OSA among Veterans with OA. It is one of the first studies to examine the prevalence of OSA among patients with OA and illustrates the importance of screening for OSA in this patient population. Results also indicated that unrecognized insomnia and OSA may be common. Given the potential for sleep problems to exacerbate disease processes, pain, and disability, and increase risks for other health problems, screening and providing evidence-based treatment for both insomnia and OSA should be a priority for providers working with individuals with OA.
